# The influence of meteorological factors and total malignant tumor health risk in Wuhu city in the context of climate change

**DOI:** 10.1186/s12889-023-15200-1

**Published:** 2023-02-16

**Authors:** Zhipeng Pan, Lingxiang Yu, Ming Shao, Yubo Ma, Yuting Cheng, Ye Wu, Shanshan Xu, Congjun Zhang, Jiansheng Zhu, Faming Pan, Guoping Sun

**Affiliations:** 1 Department of Medical Oncology, the First Affiliated Hospital of Anhui Medical University, Hefei, 230022 Anhui China; 2 Department of Epidemiology and Biostatistics, School of Public Health, Anhui Medical University, 81 Meishan Road, Hefei, 230032 Anhui China; 3 The Inflammation and Immune Mediated Diseases Laboratory of Anhui Province, Anhui Medical University, 81 Meishan Road, Hefei, 230032 Anhui China; 4 Wuhu Center for Disease Control and Prevention, Wuhu, Anhui Province China

**Keywords:** All-malignant tumors, Absolute humidity, Climate change, Short-term effect

## Abstract

With the increasing severity of the malignant tumors situation worldwide, the impacts of climate on them are receiving increasing attention. In this study, for the first time, all-malignant tumors were used as the dependent variable and absolute humidity (AH) was innovatively introduced into the independent variable to investigate the relationship between all-malignant tumors and meteorological factors. A total of 42,188 cases of malignant tumor deaths and meteorological factors in Wuhu City were collected over a 7-year (2014–2020) period. The analysis method combines distributed lagged nonlinear modeling (DLNM) as well as generalized additive modeling (GAM), with prior pre-analysis using structural equation modeling (SEM). The results showed that AH, temperature mean (T mean) and diurnal temperature range (DTR) all increased the malignant tumors mortality risk. Exposure to low and exceedingly low AH increases the malignant tumors mortality risk with maximum RR values of 1.008 (95% CI: 1.001, 1.015, lag 3) and 1.016 (95% CI: 1.001, 1.032, lag 1), respectively. In addition, low and exceedingly low T mean exposures also increased the risk of malignant tumors mortality, the maximum RR was 1.020 (95% CI: 1.006, 1.034) for low T mean and 1.035 (95% CI: 1.014, 1.058) for exceedingly low T mean. As for DTR, all four levels (exceedingly low, low, high, exceedingly high, from low to high) of exposure increased the risk of death from malignant tumors, from exceedingly low to exceedingly high maximum RR values of 1.018 (95% CI: 1.004, 1.032), 1.011 (95% CI: 1.005, 1.017), 1.006 (95% CI: 1.001, 1.012) and 1.019 (95% CI: 1.007, 1.031), respectively. The results of the stratified analysis suggested that female appear to be more sensitive to humidity, while male require additional attention to reduce exposure to high level of DTR.

## Introduction

Malignant tumor is a kind of malignant disease with very fast growth rate, which has extremely aggressive, distal spreading ability and metastatic ability [1]. In addition, cancer, as a type of malignancy, is the number one cause of premature death in China in 2019, according to a survey on global deaths [2]. Not only that, the top-ranked disease in terms of mortality among Chinese urban residents in 2020 is also malignant tumor (China Health Statistics Yearbook 2021). As climate change intensifies, the resulting effects on our health are no longer a matter of speculation [3]. However, the impact of climate change on chronic diseases such as malignant tumors is not as clear now, as the timing of chronic diseases is not closely related to exposure due to climate change. In view of this, there are relatively few studies on climate change and malignant tumors worldwide, and most studies have only explored the relationship between a particular type of tumor and environmental exposure. One literature review shows that breast, cervical, colorectal, lung and stomach cancers are most common among women, while lung, prostate, liver, colorectal and stomach cancers are most common among men [4]. In the context of the emergence of so many types of cancer, the study of malignant tumors should not be limited to one. Global trends show that malignant tumors are likely to be a major obstacle to increasing life expectancy and the major cause of death in almost every country in the world in the twenty-first century [5].

Humidity is one of the most common of the many meteorological factors, so it is often used to explore the relationship between meteorological factors and human health. In the context of global climate change, both indoor and outdoor humidity are increasing [6, 7], so the relationship between the two needs to be further explored in the context of such serious malignant tumors problems. There are already studies that point to a significant association between humidity and the development of cancers such as lung, breast, and colon cancers [8, 9]. In addition, other studies have shown that humidity may affect the development and progression of malignant tumors through a variety of ways, such as affecting the adhesion and drug resistance of tumor cells [9, 10]. However, at present, most of the research humidity indicators are selected as relative humidity (RH), which is often inappropriate in the context of epidemiology and environmental hygiene. RH is the result of a functional transformation of temperature and water vapor in the air, its size is related not only to the water vapor content in space, but also to the space temperature at that time [11], so the true moisture content of the air is not adequately reflected by it [12]. This is also why some of the environmental epidemiological studies that have included RH do not well explain its relationship with health [13, 14]. This study innovatively introduces absolute humidity (AH), which, unlike RH, is a purely physical parameter that reflects the water vapor content of the atmosphere, and is able to directly characterize the moisture content in the space unit. The unit of AH is kg/m^3^, which refers to the water vapor mass contained in each cubic meter of wet air [15], the higher the value, the higher the water vapor content in the air. There are no reports exploring the association between AH and malignant tumor mortality and there are no reports exploring meteorological factors and all-malignant tumor causes of death. To fill these gaps in the field, we included all-malignant tumor death as a dependent variable for the first time and innovatively included AH to explore the association of meteorological factors (temperature mean, AH, diurnal temperature range) with the risk of malignant tumor death. To sum up, this research is necessary in today's changing climate and increasingly challenging malignant tumors situation.

Wuhu City has four distinct seasons, and adjacent to the Yangtze River, where the humid air is year-round. There are no studies related to malignant tumors and climate in this city, so that we chose Wuhu as the study site. In addition, we control confounding factors such as contaminants with distributed lag nonlinear model (DLNM). These findings not only fill a research gap in the region, but also provide a reference for the world research on environment and all-malignant tumors.

## Materials and methods

### Basic information and overview of the study site

**Fig. 1:**
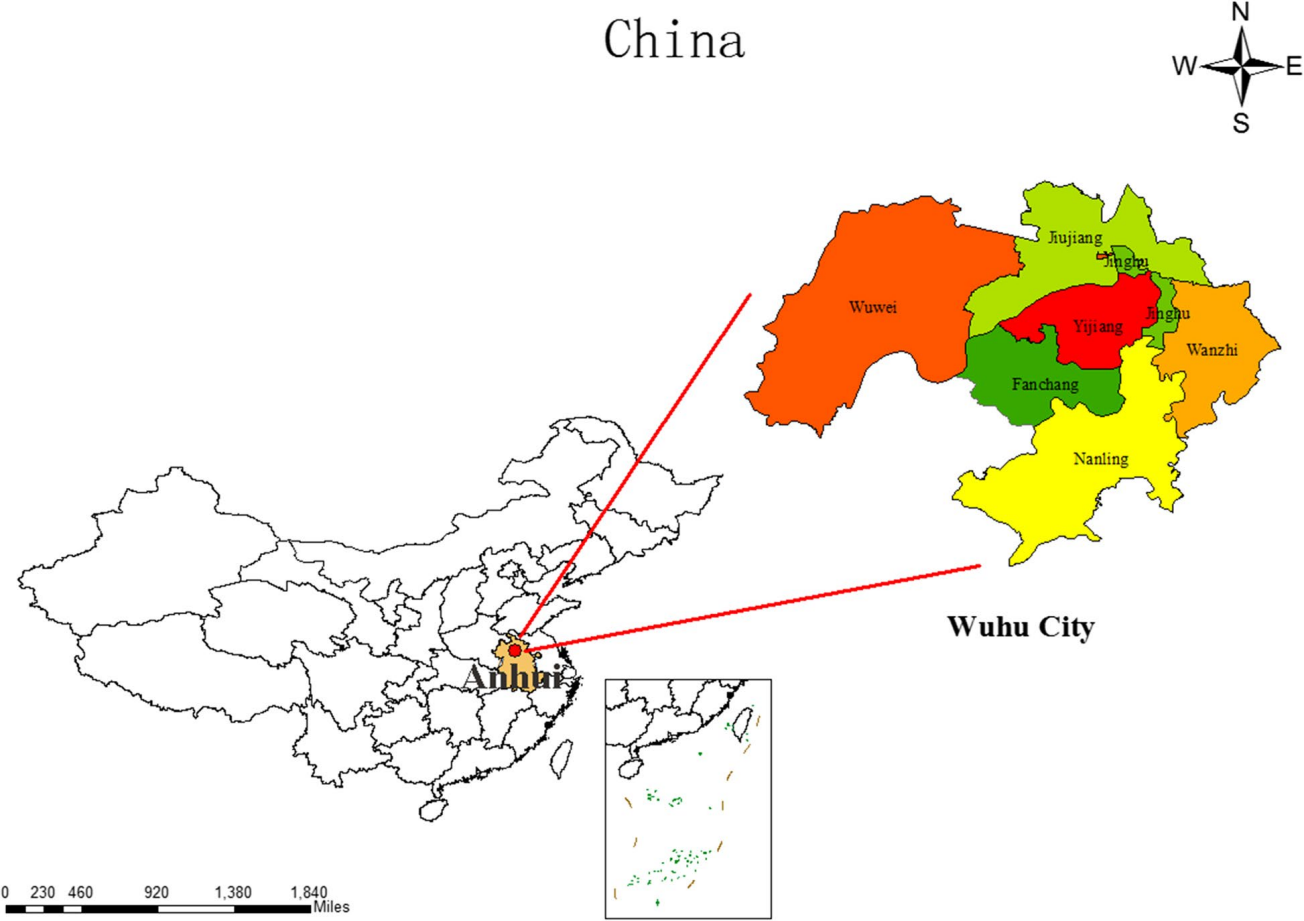
Geographical location of Wuhu City and accurate map of districts and counties

Wuhu is a prefecture-level city in East China, located in the lower reaches of the Yangtze River (Fig. 1). It is an integral part of the lower Yangtze River Plain, which can reach a total area of 6,026 square kilometers and has a humid north subtropical monsoon climate. Wuhu has excellent water transportation conditions. It is the largest freight, foreign trade and container transfer port in Anhui Province, and also a first-level port in China, which lays the foundation for a large number of population flows in Wuhu. According to the results announced by Wuhu Bureau of Statistics, the resident population of Wuhu reaches 3.672 million in 2021, the mobile population is 968,000, and the urbanization rate of resident population reaches 72.99%. In addition, according to the data released by Wuhu Meteorological Bureau, the average daily temperature and AH in Wuhu City showed a rising trend from 2014 to 2020. Compared with 2014, the average daily temperature in 2020 increased by 0.7 ℃, while the average daily AH increased by 0.5 kg/m^3^.

### Source and collection of data

All cause-of-death data for this study were provided by the Wuhu Center for Disease Control and Prevention (CDC). According to the International Classification of Diseases (ICD-10), statistics of underlying cause of deaths of all malignant tumors (C00-C99) in Wuhu City from January 1, 2014 to December 31, 2020. All death data were strictly registered by Wuhu CDC from the city's population during 2014–2020 in accordance with China's death Registration and Reporting Information Management regulations. Air pollutant data from Wuhu Environmental Monitoring Center, including the 8-h highest ozone concentration (O_3_), fine particulate matter (PM_2.5_), carbon monoxide (CO), inhaled particulate matter (PM_10_), nitrogen dioxide (NO_2_) and sulfur dioxide (SO_2_). Meteorological data from Wuhu Meteorological Bureau, including relative humidity, daily average temperature, daily maximum temperature and daily minimum temperature, where daily maximum temperature and minimum temperature are used to calculate daily average temperature difference; relative humidity and average temperature are used to calculate absolute humidity. The formula of AH is as follows:

$$\text{A}\text{H}(\text{k}\text{g}/\text{m}^3)=\frac{6.112\times e^\wedge\left[\left(17.67\times T\;mean\right)/\left(T\;mean+243.5\right)\right]\times RH\times2.1674}{\left(273.15+T\;mean\right)}$$

Where, $T mean$ represents the temperature mean and $RH$ represents the relative humidity.

### Statistical methods

We calculated the rate and number of days they were the main pollutants based on the Air Quality Index (AQI) for each pollutant. A descriptive analysis of meteorological factors, air pollutants, and daily malignant tumor deaths was performed, including maximum, minimum, mean, median (50th percentile), standard deviation, 25th percentile, 5th percentile, 75th percentile and 95th percentile. In addition, we removed the strongly correlated variables using the Spearman correlation test, and the two variables were considered to be strongly correlated when the test result was greater than 0.7 [16]. Before performing a time series analysis on the processed data, we performed a pre-analysis of them using structural equation modeling (SEM). SEM can reveal potential relationships between dependent variables and multiple independent variables, thereby uncovering indirect and direct effects between them. The model SEM has some limitations in dealing with non-linear effects, but it is still a good prior model before conducting time series [17]. The SEM formula is shown below:

$$\eta =\mathrm{\alpha }+\mathrm{\Gamma X}+\updelta$$

$\eta$ denotes the number of deaths from malignant tumors; $\alpha$ indicates the intercept distance; $\Gamma$ is the linear effect coefficient; X represents potential variables; $\delta$ means residuals. Confirmatory factor analysis was used to test the validity as well as reliability of SEM results. The results showed that the standardized root mean square residuals were 0.03 (< 0.10), the comparative fitting index was 0.97 (> 0.90), and the standard factor loading values were all higher than 0.70, indicating that the model is in good fitting state. In general, environmental exposures are complex and variable, and therefore their effects on human health are often nonlinear. However, the flexibility of the generalized additive model (GAM) can handle the nonlinear points in the environment very well. The number of daily malignant tumors deaths in this study is a small probability event, so it approximately obeys a Poisson distribution. The distributed nonlinear lag model (DLNM) is a nonlinear prediction model widely used at present. Its core idea is the cross basis. By selecting appropriate basis functions for the two dimensions of expose-response and expose-hysteresis effect, the cross basis function is obtained by calculating the tension product of the two basis functions, and then the cross basis is included in the model for analysis. DLNM is obeying Poisson distribution, so we combined it with GAM to analyze in detail the effect of climate change on population malignancy death. Under a certain pressure and a certain temperature conditions, the unit volume of air can contain water vapor is a limit. If the volume of air containing water vapor exceeds this limit, the water vapor will condense and produce precipitation, and the volume of air actually contains water vapor value, expressed by AH. AH is the result of converting temperature mean (T mean) and RH, which makes the collinearity between AH and T mean strong, so we constructed the models for both separately. The time series model of diurnal temperature range (DTR) and AH is as follows:

$$\begin{array}{l}{Y}_{t}\sim Poisson({\mu }_{t})\\Log\left({\mu }_{t}\right)= \alpha +\beta {AH}_{t,l}+\gamma {DTR}_{t,l}+ns({Pollutant}_{t},df)+ns(Time,df)+factor(Holiday)+factor(DOW)\end{array}$$

where, ${\mu }_{t}$ represents the daily number of malignant tumor deaths; $\mathrm{\alpha }$ means the intercept distance; ${AH}_{t,l}$ is the 0 ~ *l* day lag matrix of AH, *β* corresponds to the vector coefficients of AH matrix; ${DTR}_{t,l}$ is the 0 ~ *l* day lag matrix of DTR, γ corresponds to the vector coefficients of DTR matrix; where *t* is the observation time, *l* is the lag days; *ns*() represents a smooth spline function specific to DLNM; ${Pollutant}_{t}$ including O_3_, PM_2.5_, SO, NO_2_ and CO; *df* indicates the degree of freedom; $ns(Time,df)$ denotes the long-term trend and time after smooth spline function controlling for confounding effects; the holidays $factor(Holiday)$ and number of days per week $factor(DOW)$ are controlled variables. Among meteorological factors, T mean is the average value of the monitored temperature in each time period of the day, while DTR represents the difference between the maximum temperature and the minimum temperature of the day. Although there is a certain correlation between the two, the actual correlation is not strong, so they can be included in the same model. The daily malignant tumors mortality time series models for DTR and T mean are as follows:

$$\begin{array}{l}Y_t\sim Poisson(\mu_t)\\Log\left(\mu_t\right)=\alpha+\delta{T\;mean}_{t,l}+\varepsilon{DTR}_{t,l}+ns({Pollutant}_t,df)+ns(Time,df)+factor(Holiday)+factor(DOW)\end{array}$$

DLNM produces a T mean matrix ${T mean}_{t,l}$ with 0 ~ *l* lag days; *δ* denotes the vector coefficient of the T mean matrix; *ε* is the vector coefficient of the DTR matrix ${DTR}_{t,l}$. The evaluation of the degree of fit and freedom of these two models is done with the Akakchi information criterion (AIC) and residual analysis. Since there are no reports of meteorological factors associated with all-malignant tumors, we reviewed similar literature and determined the lag days as 2 weeks (14 days) [18]. Using the natural cubic spline function (NCSF) as a natural smooth spline function for meteorological factors and pollutants, the AIC value is lowest when the degree of freedom is 3*df*. Moreover, the secular trend as well as the temporal distribution is controlled by the NCSF, with 7 being the annual degree of freedom ($Time,df=7*year$). NCSF was selected as the cross basis for fitting, in order to connect the obtained two-dimensional functions and make the resulting curve more smooth. We used RR and its 95% CI to depict cumulative and single-day risk results for death from malignant tumors. Finally, the 50th percentile of meteorological factors was used as a reference to classify meteorological factors into four categories: high (75th), exceedingly high (95th), low (25th) and exceedingly low (5th).

### Software usage

Map of Wuhu City, Anhui Province, China by the software of ArcMap Desktop (10.7.0.10450 version). Descriptive analyses were performed with SPSS 23.0, and we performed the remaining statistical analyses with the software of RGui (version 4.1.2). The matching of meteorological and pollutant models in time series is implemented with "spline" and "DLNM" packages. In addition, SEM is run usinssg the "lavaan" package, and variable-related analysis is done using the "PerformanceAnalytics" package. The criteria for determining with bilateral statistical differences were *p*-value < 0.05.

## Results

### Descriptive summary

The distributions of daily malignant tumors deaths, major pollutants and meteorological factors in Wuhu, China, 2014–2020 are shown in Table 1. We collected a total of 42,188 malignant tumors deaths over a period of 2557 days (7 years), with an average of 16.50 deaths per day. There were 24,921 (59.07%) cases of digestive system tumors, 10,543 (24.99%) cases of respiratory system tumors, 1,634 (3.87%) cases of lymphatic and hematopoietic tumors, and 5,090 (12.07%) cases of other types of tumors. Among all deaths, there were 28,481 cases (67.51%) in male, 13,707 cases (32.49%) in female, 12,688 cases (30.07%) in the 0–65 age group, and 29,500 cases (69.93%) in the ≥ 65 age group. Among them, the ratio of men to women is about 2:1, and the ratio of young people (0–65 years old) to the elderly (≥ 65 years old) is about 1:2.5. Wuhu is located in the eastern part of Anhui Province, downstream of the Yangtze River, with a daily average DTR of 8.66 ℃ (range: 1.00 ℃—24.00 ℃), AH of 12.38 g/m^3^ (range: 1.43 g/m^3^—26.11 g/m^3^), T mean of 16.96 ℃ (range: -6.96 ℃—35.04 ℃) and RH of 76.68% (range: 35.38%—100.00%). The average concentrations of each air pollutant were 50.01 μg/m^3^ (PM_2.5_, range: 4.00 μg/m^3^—302.00 μg/m^3^), 73.22 μg/m^3^ (PM_10_, range: 0.00 μg/m^3^—367.00 μg/m^3^), 14.95 μg/m^3^ (SO_2_, range: 3.00 μg/m^3^—104.00 μg/m^3^), 37.70 μg/m^3^ (NO_2_, range: 8.00 μg/m^3^—126.00 μg/m^3^), 963.21 μg/m^3^ (CO, range: 240.00 μg/m^3^—2670.00 μg/m^3^), 60.44 μg/m^3^ (O_3_, range: 2.00 μg/m^3^—198.00 μg/m^3^). In these 7 years, PM_2.5_ as the main air pollutant accounted for 1103 days (43.14%), PM_10_ for 602 days (23.54%), NO_2_ for 445 days (17.40%), O_3_ for 394 days (15.41%), SO_2_ for 9 days (0.35%), and CO for 4 days (0.16%).

**Table 1:** Statistics of daily death toll, meteorological conditions and air pollutants in Wuhu (2014 to 2020)

Variables	Counts (%)	$\mathrm{Mean}$ $\pm$ $\mathrm{SD}$	Centiles						
			Minimum	*P*_5_	*P*_25_	Median	*P*_75_	*P*_95_	Maximum
**Malignant tumor**									
Total	42,188 (100.00)	16.50 ± 5.11	3	9	13	16	20	25	36
**Tumor classification**									
Digestive	24,921 (59.07)	9.75 ± 3.62	1	4	7	9	12	16	24
Respiratory	10,543 (24.99)	4.12 ± 2.22	0	1	3	4	5	8	14
Lymphatic, hematopoietic	1634 (3.87)	16.50 ± 5.11	0	0	0	0	1	2	5
Other	5090 (12.07)	16.50 ± 5.11	0	0	1	2	3	5	10
**Gender and age**									
Male	28,481 (67.51)	11.14 ± 3.93	1	5	8	11	14	18	25
Female	13,707 (32.49)	5.36 ± 2.55	0	2	4	5	7	10	16
0–65 years	12,688 (30.07)	4.96 ± 2.37	0	1	3	5	7	9	16
≥ 65 years	29,500 (69.93)	11.54 ± 4.16	1	5	9	11	14	19	30
**Meteorological conditions**									
DTR (℃)	-	8.66 ± 4.23	1.00	2.00	5.00	9.00	12.00	16.00	24.00
AH (g/m^3^)	-	12.38 ± 6.43	1.43	3.98	6.56	11.27	17.71	23.62	26.11
Tmean (℃)	-	16.96 ± 9.09	-6.96	2.41	8.75	17.50	24.29	30.55	35.04
RH (%)	-	76.68 ± 12.32	35.38	55.96	67.96	76.88	86.00	93.50	100.00
**Air pollutants**									
PM_2.5_ (μg/m^3^)	1103 (43.14)	50.01 ± 33.31	4.00	15.00	27.00	42.00	64.00	91.40	302.00
PM_10_ (μg/m^3^)	602 (23.54)	73.22 ± 42.85	0.00	25.00	43.00	63.00	94.00	126.00	367.00
SO_2_ (μg/m^3^)	9 (0.35)	14.95 ± 10.60	3.00	5.00	8.00	12.00	18.00	27.00	104.00
NO_2_ (μg/m^3^)	445 (17.40)	37.70 ± 17.52	8.00	15.00	25.00	35.00	47.00	63.00	126.00
CO (μg/m^3^)	4 (0.16)	963.21 ± 322.11	240.00	560.00	740.00	900.00	1130.00	1400.00	2670.00
O_3_ (μg/m^3^)	394 (15.41)	60.44 ± 34.54	2.00	19.00	36.00	51.00	78.00	109.00	198.00

*Abbreviations*: *SD* Standard deviation, *DTR* Diurnal temperature range, *AH* Absolute humidity, *Tmean* temperature mean, *PM*_*2.5*_ Particulate matter ≤ 2.5 μm in aerodynamic diameter, *PM*_*10*_ Particulate matter ≤ 10 μm in aerodynamic diameter, *SO*_*2*_ Sulfur dioxide, *NO*_*2*_ Nitrogen dioxide, *CO* Carbon monoxide, *O*_*3*_ Ozone; Counts of Air pollutants: number and proportion of days with each air pollutant as a daily major air pollutant

### SEM and relevance analysis

**Fig. 2:**
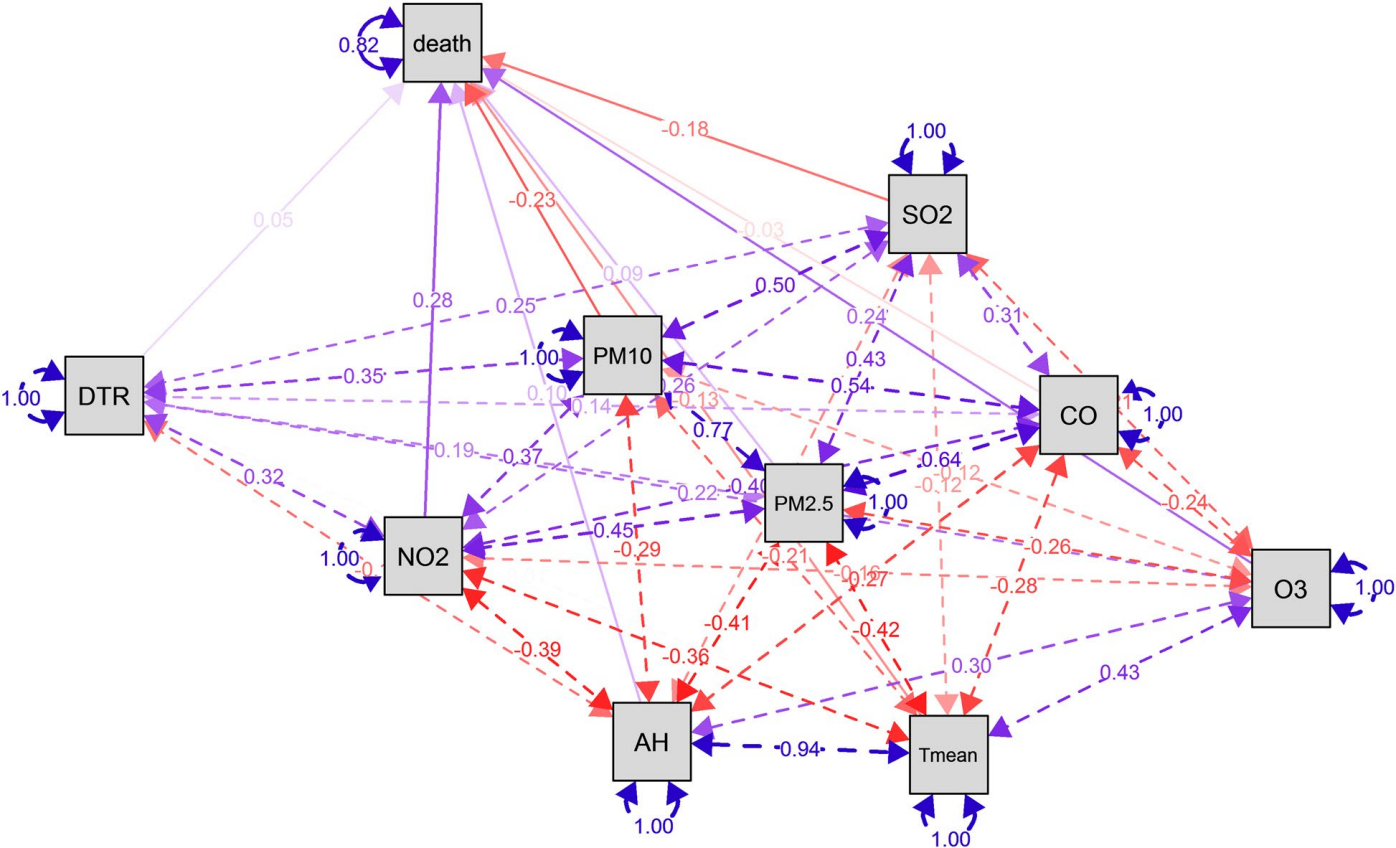
SEM analysis of the direct and indirect climate effects on malignant tumors mortality

**Fig. 3:**
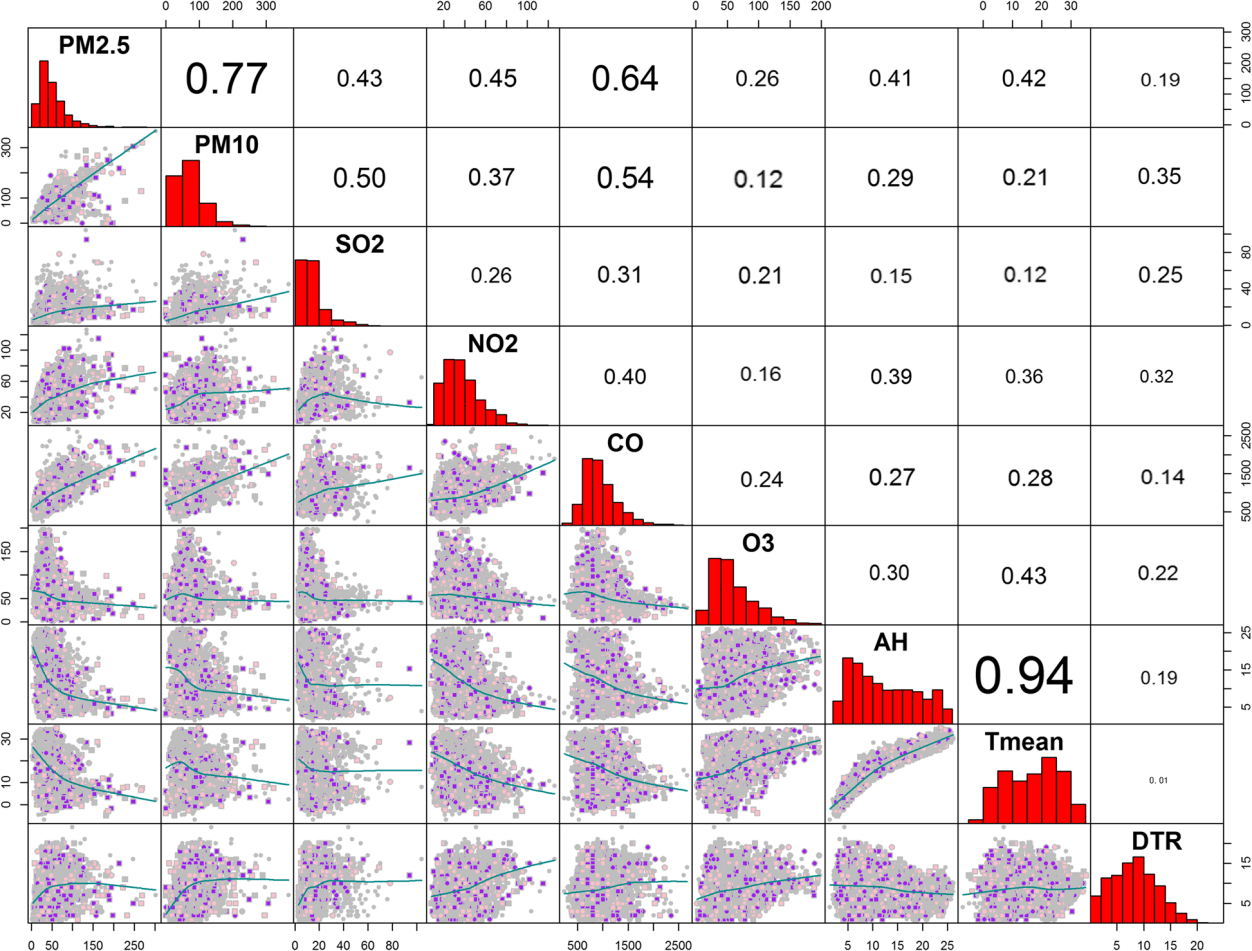
Spearman’s correlation coefficients meteorological factors and atmospheric pollutants: Spearman’s correlation coefficients at the top, distribution plot at the middle and scatter plot at the bottom

We conducted SEM on the collected information before analyzing the data (Fig. 2). The blue line shows positive correlation, the red line shows negative correlation, the solid line shows direct correlation between independent variables and dependent variables, and the dotted line shows correlation between independent variables. The results showed that air pollutants and meteorological factors could have indirect or direct effects on the risk of malignant tumors death, with AH, DTR, NO_2_, O_3_ and PM_2.5_ having a positive effect on disease risk, while T mean, PM_10_, SO_2_ and CO had a negative effect on disease risk. Although SEM can reveal linear relationships among variables well, it has limited ability to capture nonlinear effects, so we need to be cautious when using SEM to analyze the relationships among variables. It is now widely believed that the environment has a limited impact on the human body and therefore the relationship between the two is nonlinear. From the results, it follows that the SEM results are not exactly the same as the time series results, but its results can still be used as a reference before the start of the time series model [17]. The outcomes of Spearman correlation analysis of air pollutants and meteorological factors are shown in Fig. 3. There were significant positive correlations between PM_2.5_ and PM_10_, and between AH and T mean (*P* < 0.001, *r*_*s*_ > 0.7). Both AH and T mean were negatively correlated with NO_2_, PM_2.5_, SO_2_, PM_10_ and CO, and positively correlated with O_3_ (*P* < 0.001). DTR was positively correlated with NO_2_, PM_2.5_, SO_2_, O_3_, PM_10_ and CO (*P* < 0.001).

### The overall effect of AH, T mean and DTR on the risk of malignant tumors death

**Fig. 4:**
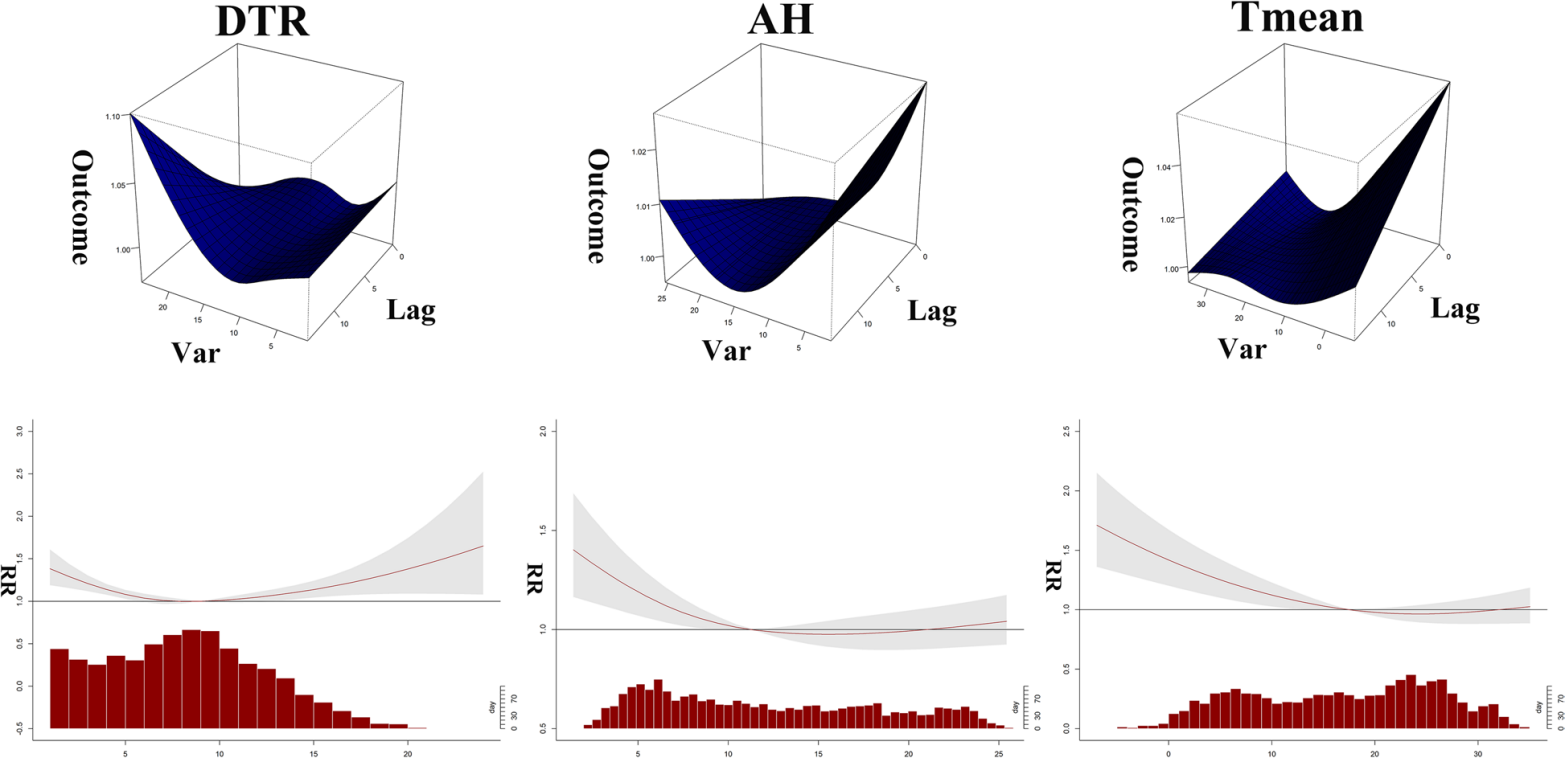
The 3D graph, and overall exposure–response association curve between DTR, AH, Tmean and malignant tumors mortality

The exposure–response relationships of AH, T mean and DTR at different values with the total risk of malignant tumors death are shown in Fig. 4. The results showed that AH, T mean and DTR can all increase the risk of death from malignant tumors. Using the median AH of 11.27 g/m^3^ as a reference, in the single-day lag effects model, low AH persists for 10 days and exceedingly low AH persists for 12 days (*P* < 0.05), with both decreasing trends and maximum RR values were 1.008 (95% CI: 1.001, 1.015, lag 3) and 1.016 (95% CI: 1.001, 1.032, lag 1). In the cumulative daily hysteresis effects model, the effects of both exceedingly low and low AH gradually increase, with the greatest RR value was 1.120 (95% CI: 1.036, 1.211, lag 0–14) for exceedingly low AH and 1.245 (95% CI: 1.096, 1.413, lag 0–14) for low. (Table 2).

**Table 2:** Relative risk (RR) of malignant tumors daily death for specific AH on different lag days

	single-day lag					cumulative-day lag			
lag	5th percentile	25th percentile	75th percentile	95th percentile	lag	5th percentile	25th percentile	75th percentile	95th percentile
0	1.016 (0.998,1.035)	1.008 (0.997,1.018)	0.998 (0.985,1.011)	0.996 (0.976,1.016)	0–0	1.016 (0.998,1.035)	1.008 (0.997,1.018)	0.998 (0.985,1.011)	0.996 (0.976,1.016)
1	1.016 (1.001,1.032) *	1.008 (0.998,1.017)	0.998 (0.987,1.010)	0.996 (0.979,1.015)	0–1	1.033 (0.998,1.068)	1.016 (0.996,1.036)	0.996 (0.973,1.021)	0.992 (0.955,1.031)
2	1.016 (1.001,1.030) *	1.008 (0.999,1.016)	0.998 (0.988,1.008)	0.997 (0.982,1.013)	0–2	1.049 (1.000,1.101) *	1.023 (0.995,1.052)	0.995 (0.961,1.029)	0.989 (0.938,1.044)
3	1.016 (1.003,1.029) *	1.008 (1.001,1.015) *	0.998 (0.990,1.007)	0.998 (0.985,1.012)	0–3	1.065 (1.003,1.132) *	1.031 (0.996,1.068)	0.993 (0.951,1.036)	0.988 (0.924,1.056)
4	1.015 (1.004,1.027) *	1.008 (1.001,1.014) *	0.998 (0.991,1.006)	0.999 (0.988,1.010)	0–4	1.082 (1.007,1.162) *	1.039 (0.997,1.083)	0.991 (0.943,1.042)	0.987 (0.913,1.067)
5	1.015 (1.005,1.025) *	1.008 (1.002,1.014) *	0.999 (0.992,1.005)	1.000 (0.990,1.009)	0–5	1.098 (1.013,1.190) *	1.047 (0.999,1.098)	0.990 (0.936,1.047)	0.986 (0.904,1.076)
6	1.015 (1.006,1.024) *	1.008 (1.002,1.013) *	0.999 (0.993,1.005)	1.001 (0.993,1.009)	0–6	1.115 (1.020,1.218) *	1.055 (1.002,1.111) *	0.989 (0.930,1.051)	0.987 (0.899,1.084)
7	1.015 (1.006,1.023) *	1.008 (1.002,1.013) *	0.999 (0.993,1.004)	1.002 (0.994,1.009)	0–7	1.131 (1.028,1.244) *	1.063 (1.005,1.124) *	0.987 (0.925,1.054)	0.989 (0.896,1.091)
8	1.014 (1.006,1.023) *	1.008 (1.002,1.013) *	0.999 (0.993,1.005)	1.002 (0.995,1.010)	0–8	1.147 (1.038,1.269) *	1.071 (1.010,1.137) *	0.986 (0.921,1.056)	0.991 (0.895,1.097)
9	1.014 (1.005,1.023) *	1.008 (1.002,1.013) *	0.999 (0.992,1.005)	1.003 (0.995,1.012)	0–9	1.164 (1.047,1.293) *	1.079 (1.014,1.149) *	0.985 (0.917,1.058)	0.994 (0.896,1.103)
10	1.014 (1.004,1.024) *	1.008 (1.001,1.014) *	0.999 (0.991,1.006)	1.004 (0.994,1.014)	0–10	1.180 (1.058,1.316) *	1.087 (1.019,1.161) *	0.984 (0.914,1.059)	0.998 (0.899,1.108)
11	1.014 (1.003,1.025) *	1.007 (1.001,1.014) *	0.999 (0.990,1.008)	1.005 (0.993,1.017)	0–11	1.196 (1.068,1.339) *	1.096 (1.024,1.173) *	0.983 (0.911,1.061)	1.003 (0.903,1.114)
12	1.014 (1.001,1.026) *	1.007 (1.000,1.015) *	0.999 (0.989,1.009)	1.006 (0.992,1.020)	0–12	1.212 (1.079,1.362) *	1.104 (1.028,1.185) *	0.982 (0.907,1.062)	1.009 (0.907,1.121)
13	1.013 (0.999,1.028)	1.007 (0.999,1.016)	0.999 (0.988,1.010)	1.007 (0.990,1.023)	0–13	1.228 (1.088,1.387) *	1.112 (1.032,1.198) *	0.981 (0.903,1.065)	1.015 (0.912,1.131)
14	1.013 (0.997,1.029)	1.007 (0.998,1.017)	0.999 (0.987,1.012)	1.007 (0.989,1.027)	0–14	1.245 (1.096,1.413) *	1.120 (1.036,1.211) *	0.980 (0.898,1.070)	1.023 (0.915,1.144)

The table records use the mean of RR values and 95% confidence intervals; *P < 0.05

Table 3 records the relationship between different levels of T mean and daily malignant tumors mortality. Using median 17.50 ℃ as a reference, the effect of low and exceedingly low T mean in the single-day hysteresis effect model began at lag 0 and ended at lag 8 and lag 10, respectively. The maximum RR was 1.020 (95% CI: 1.006, 1.034) for low T mean and 1.035 (95% CI: 1.014, 1.058) for exceedingly low T mean, both on the day of the lag (lag 0). In the cumulative daily hysteresis effect model, the significant effects of low and exceedingly low T mean persisted the entire lag research cycle (lag 0–0 until lag 0–14), but the difference was that the effect trend of low T mean increased first and then decreased, while that of exceedingly low continued to increase. The maximum RR values for low and exceedingly low T mean were 1.153 (95% CI: 1.048, 1.268) and 1.333 (95% CI: 1.151, 1.545), respectively.

**Table 3:** Relative risk of malignant tumors daily death for specific T mean on different lag days

	single-day lag					cumulative-day lag			
lag	5th percentile	25th percentile	75th percentile	95th percentile	lag	5th percentile	25th percentile	75th percentile	95th percentile
0	1.035 (1.014,1.058) *	1.020 (1.006,1.034) *	0.993 (0.983,1.004)	0.999 (0.979,1.018)	0–0	1.035 (1.014,1.058) *	1.020 (1.006,1.034) *	0.993 (0.983,1.004)	0.999 (0.979,1.018)
1	1.033 (1.014,1.053) *	1.018 (1.006,1.031) *	0.994 (0.984,1.004)	0.999 (0.982,1.016)	0–1	1.070 (1.027,1.114) *	1.039 (1.011,1.067) *	0.987 (0.967,1.008)	0.997 (0.961,1.035)
2	1.031 (1.013,1.049) *	1.017 (1.006,1.028) *	0.995 (0.986,1.003)	0.999 (0.984,1.014)	0–2	1.103 (1.041,1.168) *	1.056 (1.017,1.097) *	0.982 (0.954,1.011)	0.996 (0.946,1.049)
3	1.029 (1.013,1.044) *	1.015 (1.005,1.026) *	0.995 (0.988,1.003)	0.999 (0.986,1.012)	0–3	1.134 (1.055,1.220) *	1.073 (1.023,1.125) *	0.977 (0.943,1.013)	0.995 (0.933,1.062)
4	1.026 (1.013,1.040) *	1.014 (1.005,1.023) *	0.996 (0.989,1.002)	0.999 (0.988,1.010)	0–4	1.164 (1.069,1.268) *	1.088 (1.028,1.150) *	0.973 (0.933,1.015)	0.994 (0.922,1.072)
5	1.024 (1.012,1.036) *	1.012 (1.005,1.020) *	0.996 (0.991,1.002)	0.999 (0.990,1.008)	0–5	1.192 (1.082,1.313) *	1.101 (1.034,1.173) *	0.970 (0.925,1.017)	0.993 (0.913,1.080)
6	1.022 (1.011,1.032) *	1.011 (1.004,1.018) *	0.997 (0.992,1.002)	0.999 (0.991,1.007)	0–6	1.218 (1.095,1.354) *	1.113 (1.039,1.193) *	0.967 (0.918,1.018)	0.993 (0.907,1.087)
7	1.019 (1.009,1.029) *	1.009 (1.003,1.016) *	0.998 (0.993,1.003)	0.999 (0.992,1.007)	0–7	1.241 (1.108,1.391) *	1.124 (1.044,1.210) *	0.964 (0.913,1.019)	0.992 (0.901,1.091)
8	1.017 (1.007,1.027) *	1.008 (1.001,1.015) *	0.998 (0.993,1.003)	0.999 (0.991,1.007)	0–8	1.262 (1.120,1.423) *	1.133 (1.048,1.225) *	0.963 (0.908,1.020)	0.991 (0.898,1.094)
9	1.015 (1.005,1.025) *	1.007 (0.999,1.014)	0.999 (0.993,1.005)	0.999 (0.990,1.009)	0–9	1.281 (1.130,1.452) *	1.140 (1.051,1.237) *	0.961 (0.905,1.021)	0.991 (0.895,1.096)
10	1.013 (1.001,1.024) *	1.005 (0.997,1.013)	0.999 (0.993,1.006)	1.000 (0.989,1.010)	0–10	1.297 (1.140,1.476) *	1.146 (1.053,1.247) *	0.961 (0.903,1.023)	0.990 (0.893,1.097)
11	1.010 (0.998,1.023)	1.004 (0.994,1.013)	1.000 (0.992,1.008)	1.000 (0.987,1.012)	0–11	1.311 (1.147,1.497) *	1.150 (1.054,1.255) *	0.961 (0.901,1.025)	0.990 (0.892,1.098)
12	1.008 (0.994,1.023)	1.002 (0.991,1.013)	1.001 (0.992,1.009)	1.000 (0.985,1.015)	0–12	1.321 (1.152,1.515) *	1.152 (1.052,1.262) *	0.961 (0.899,1.028)	0.989 (0.890,1.100)
13	1.006 (0.990,1.022)	1.001 (0.989,1.013)	1.001 (0.991,1.011)	1.000 (0.983,1.017)	0–13	1.329 (1.153,1.531) *	1.153 (1.048,1.268) *	0.962 (0.897,1.033)	0.989 (0.886,1.104)
14	1.004 (0.985,1.022)	0.999 (0.986,1.013)	1.002 (0.991,1.013)	1.000 (0.981,1.019)	0–14	1.333 (1.151,1.545) *	1.152 (1.042,1.274) *	0.964 (0.894,1.039)	0.989 (0.881,1.110)

The table records use the mean of RR values and 95% confidence intervals; *P < 0.05

The relationship between DTR and daily malignant tumors mortality risk is shown in Table 4. It is interesting to note that DTR exposure at all four levels increased the malignant tumors mortality risk in the single-day hysteresis effect model. Exceedingly high, low and exceedingly low DTR exposures had more days of effect on malignant tumors deaths, lasting 10, 11 and 15 days, respectively, while high DTR exposures lasted only 6 days. In addition, the maximum RR values for exceedingly high, low and exceedingly low DTR were all found in lag 14, the values were 1.019 (95% CI: 1.007, 1.031), 1.011 (95% CI: 1.005, 1.017) as well as 1.018 (95% CI: 1.004, 1.032); the maximum RR value for high DTR was found in lag 1, with a value of 1.006, and the 95% CI was 1.001 to 1.012. In the cumulative daily lagged effects model, only high DTR exposure did not observe a significant effect on malignant tumors mortality. Maximum RR value for high DTR exposure was 1.176 (95% CI: 1.060, 1.305, lag 0–14), low DTR exposure was significant from lag 0–11 with the greatest RR value was 1.080 (95% CI: 1.029, 1.134, lag 0–14), and exceedingly low DTR was first significant at lag 0–0 with the greatest RR value was 1.291 (95% CI: 1.156, 1.441, lag 0–14). The significant effects of all three levels of DTR exposure showed a gradual increase.

**Table 4:** Relative risk of malignant tumors daily death for specific DTR on different lag days

	single-day lag					cumulative-day lag			
lag	5th percentile	25th percentile	75th percentile	95th percentile	lag	5th percentile	25th percentile	75th percentile	95th percentile
0	1.016 (1.002,1.031) *	0.999 (0.993,1.006)	1.006 (0.999,1.013)	1.003 (0.991,1.015)	0–0	1.016 (1.002,1.031) *	0.999 (0.993,1.006)	1.006 (0.999,1.013)	1.003 (0.991,1.015)
1	1.017 (1.004,1.029) *	1.000 (0.994,1.006)	1.006 (1.001,1.012) *	1.004 (0.993,1.015)	0–1	1.033 (1.006,1.061) *	0.999 (0.987,1.012)	1.012 (0.999,1.026)	1.006 (0.984,1.030)
2	1.017 (1.005,1.028) *	1.001 (0.996,1.006)	1.005 (1.000,1.011) *	1.005 (0.995,1.015)	0–2	1.050 (1.012,1.091) *	1.000 (0.983,1.018)	1.018 (0.999,1.037)	1.011 (0.979,1.045)
3	1.017 (1.007,1.027) *	1.002 (0.997,1.006)	1.005 (1.000,1.010) *	1.006 (0.997,1.015)	0–3	1.068 (1.018,1.120) *	1.002 (0.980,1.024)	1.023 (0.999,1.047)	1.018 (0.976,1.061)
4	1.017 (1.008,1.026) *	1.003 (0.999,1.007) *	1.004 (1.000,1.009) *	1.007 (0.999,1.016)	0–4	1.086 (1.027,1.149) *	1.005 (0.979,1.031)	1.027 (0.999,1.057)	1.025 (0.976,1.077)
5	1.017 (1.009,1.025) *	1.003 (1.000,1.007) *	1.004 (1.000,1.008) *	1.009 (1.001,1.016) *	0–5	1.104 (1.036,1.177) *	1.008 (0.979,1.038)	1.031 (0.999,1.065)	1.034 (0.977,1.094)
6	1.017 (1.009,1.025) *	1.004 (1.001,1.008) *	1.004 (1.000,1.007) *	1.010 (1.003,1.017) *	0–6	1.123 (1.047,1.205) *	1.012 (0.980,1.045)	1.035 (0.999,1.072)	1.044 (0.981,1.111)
7	1.017 (1.010,1.025) *	1.005 (1.002,1.008) *	1.003 (0.999,1.007)	1.011 (1.004,1.018) *	0–7	1.143 (1.059,1.233) *	1.018 (0.983,1.053)	1.038 (0.999,1.079)	1.055 (0.986,1.130)
8	1.017 (1.010,1.025) *	1.006 (1.003,1.009) *	1.003 (0.999,1.006)	1.012 (1.005,1.019) *	0–8	1.162 (1.072,1.260) *	1.024 (0.987,1.062)	1.041 (0.999,1.085)	1.068 (0.993,1.149)
9	1.017 (1.009,1.026) *	1.007 (1.003,1.010) *	1.002 (0.998,1.006)	1.013 (1.006,1.021) *	0–9	1.182 (1.086,1.288) *	1.031 (0.992,1.071)	1.043 (0.999,1.089)	1.082 (1.001,1.170) *
10	1.017 (1.008,1.027) *	1.008 (1.004,1.012) *	1.002 (0.997,1.006)	1.014 (1.006,1.023) *	0–10	1.203 (1.100,1.316) *	1.039 (0.998,1.081)	1.045 (0.998,1.094)	1.098 (1.011,1.192) *
11	1.018 (1.008,1.028) *	1.009 (1.004,1.013) *	1.001 (0.996,1.006)	1.016 (1.007,1.025) *	0–11	1.224 (1.115,1.345) *	1.047 (1.004,1.092) *	1.046 (0.997,1.098)	1.115 (1.022,1.216) *
12	1.018 (1.006,1.029) *	1.009 (1.005,1.014) *	1.001 (0.995,1.006)	1.017 (1.007,1.027) *	0–12	1.246 (1.129,1.375) *	1.057 (1.012,1.105) *	1.047 (0.995,1.101)	1.134 (1.034,1.243) *
13	1.018 (1.005,1.030) *	1.010 (1.005,1.016) *	1.000 (0.994,1.006)	1.018 (1.007,1.029) *	0–13	1.268 (1.143,1.407) *	1.068 (1.020,1.118) *	1.047 (0.993,1.105)	1.154 (1.047,1.272) *
14	1.018 (1.004,1.032) *	1.011 (1.005,1.017) *	1.000 (0.993,1.006)	1.019 (1.007,1.031) *	0–14	1.291 (1.156,1.441) *	1.080 (1.029,1.134) *	1.047 (0.990,1.108)	1.176 (1.060,1.305) *

The table records use the mean of RR values and 95% confidence intervals; *P < 0.05

### Stratified analysis of age and gender

**Fig. 5:**
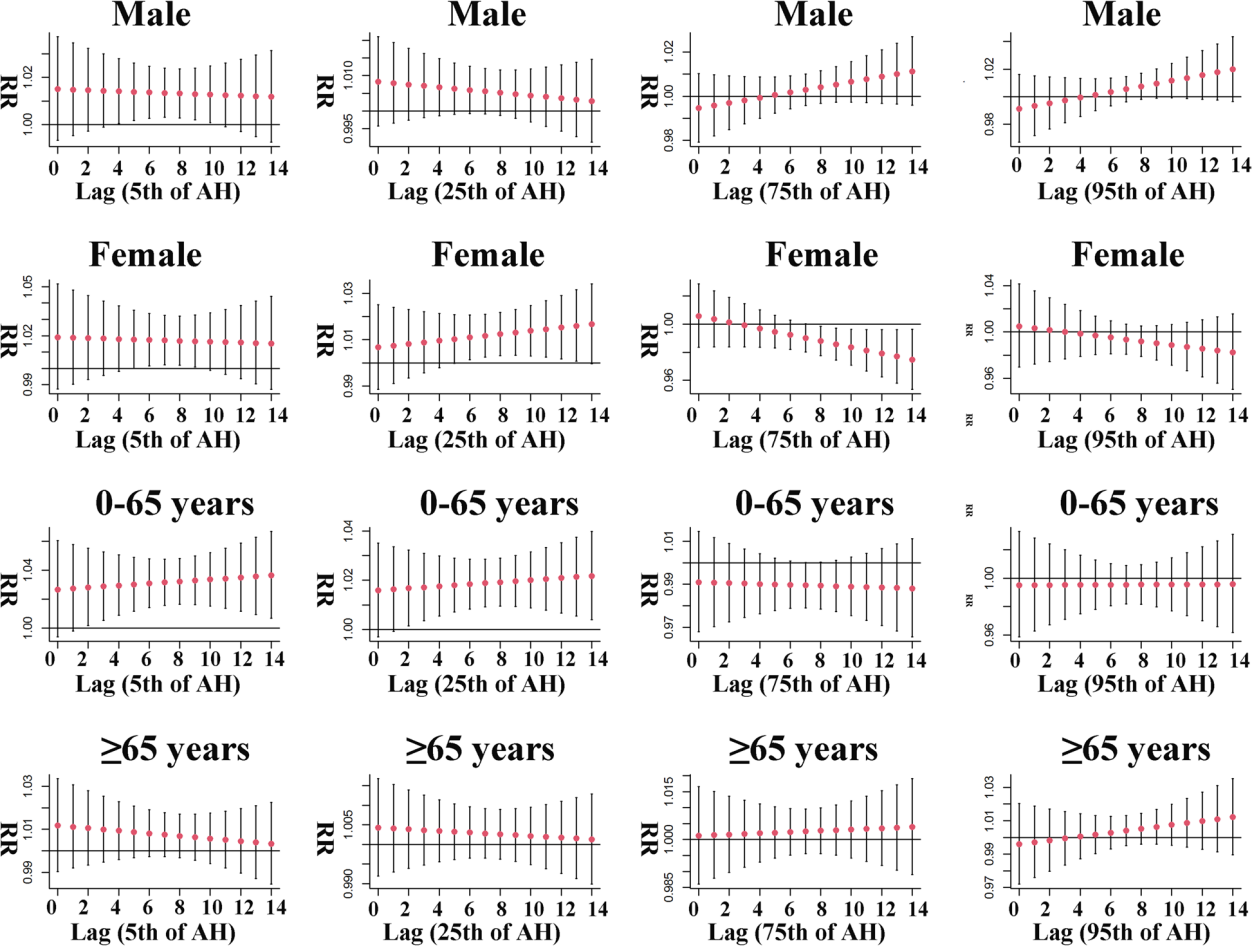
Gender and age stratified lag effect of AH on malignant tumor mortality at various lag days

Figure 5 shows the stratified analysis results of AH by age and gender. The results indicated significant effects of AH for female, male and the young, but not for the elderly. In the female population, both low and exceedingly low AH exposure increased the risk of malignant tumors death, with maximum RR values occurring at lag 13 (RR: 1.016, 95% CI: 1.001, 1.031) and lag 5 (RR: 1.018, 95% CI: 1.001, 1.036), lasting 8 and 5 days respectively. Interestingly, we discovered that high AH exposure reduced the malignant tumors mortality risk in female, with a significant effect decreasing consistently from lag 8, with a minimum RR was 0.975 (95% CI: 0.953, 0.996, lag 14). Compared to the female, male risk of malignant tumors death was increased only at exceedingly low AH exposure, with a significant risk effect starting at lag 4 and continuing until lag 10, with the greatest RR value was 1.014, the 95% CI was 1.001 to 1.028, occurring at lag 4. In the age-stratified analysis, low and exceedingly low AH exposure augment the malignant tumors mortality risk in young people, which was similar to the results for female. Maximum RR value of 1.022 (95% CI: 1.004, 1.040) for low AH exposure and 1.036 (95% CI: 1.007, 1.067) for exceedingly low AH exposure. No significant risk effects were found among the elderly population.

**Fig. 6:**
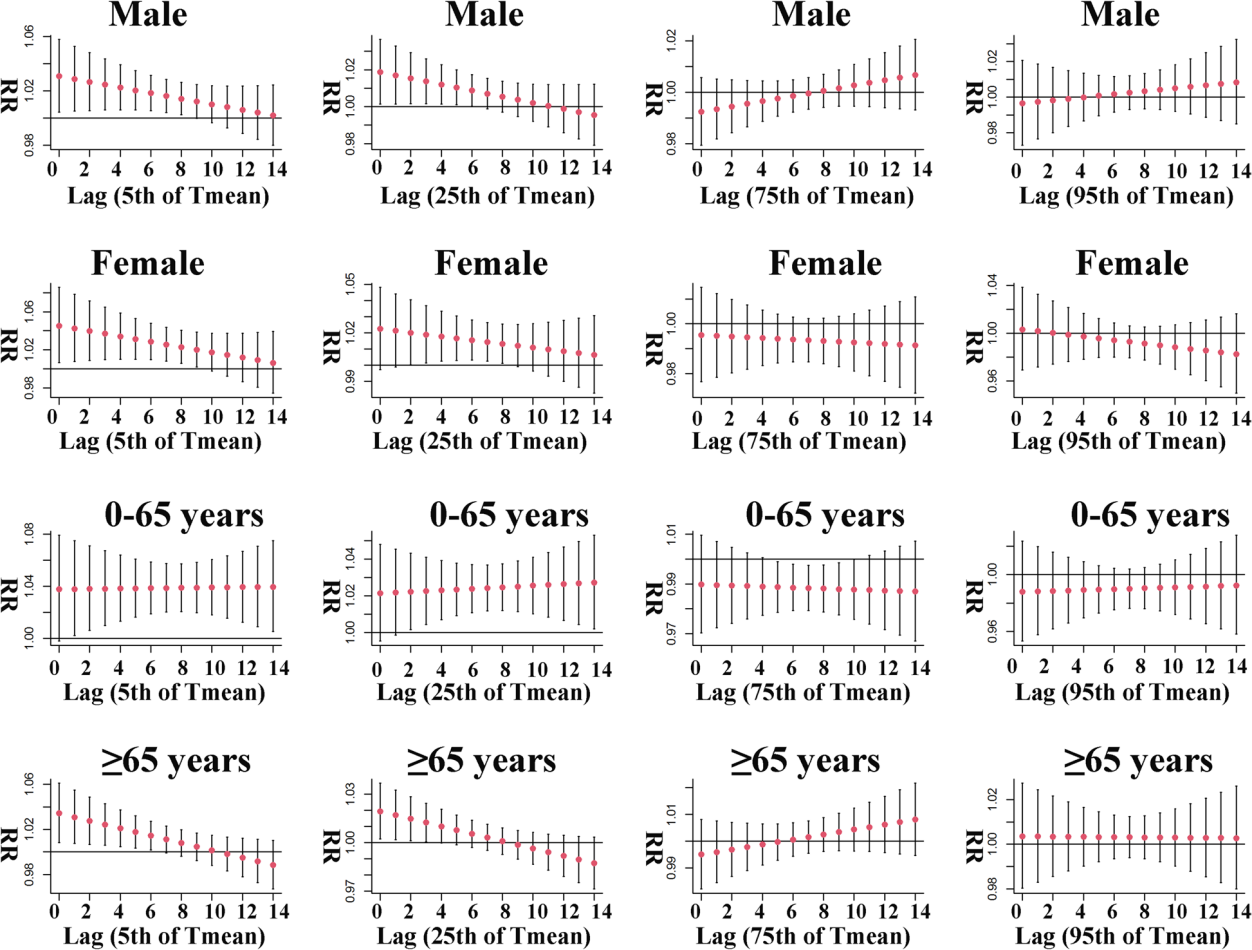
Gender and age stratified lag effect of T mean on malignant tumor mortality at various lag days

The stratified analysis of T mean showed that short-term exposure to T mean significantly affected the risk of malignant tumors death in male, female, the young and the elderly to varying degrees (Fig. 6). First, in the male population, low and exceedingly low T mean exposure increased their risk of malignant tumors death, with both levels of exposure showing maximum RR values of 1.019 (95% CI: 1.001, 1.036) and 1.031 (95% CI: 1.004, 1.058) on the day of exposure (lag 0) respectively, and decreasing thereafter, with significant effects lasting for 7 and 9 days respectively. Second, low and exceedingly low T mean exposure similarly increased the risk of malignant tumors death in female, and unlike in male, the significant effect of low T mean exposure was first seen in lag 2. The maximum RR values for female at low and exceedingly low T mean short-term exposures were 1.020 (95% CI: 1.001, 1.040, lag 2) and 1.045 (95% CI: 1.007, 1.086, lag 0), respectively. Third, we found an interesting phenomenon in the results of the analysis of young people, that their risk of malignant tumors death was progressively increased under short-term exposure to low and exceedingly low T mean, which was contrary to male, female and elderly people, and this risk in young people under high T mean exposure was reduced. Therefore, the maximum risk effect of young people under low and exceedingly low T mean exposure occurred in lag 14, with RR of 1.027 (95% CI: 1.002, 1.053) and 1.040 (95% CI: 1.006, 1.075), respectively, and the minimum RR was 0.988 (95% CI: 0.976, 0.999, lag 10) under high T mean exposure. Finally, the risk of malignant tumors death in elderly people at both low and exceedingly low T mean exposures was highest on the day of lag (lasting 4 and 7 days, respectively), with maximum RR values of 1.019 (95% CI: 1.002, 1.037) and 1.034 (95% CI: 1.008, 1.061), respectively.

**Fig. 7:**
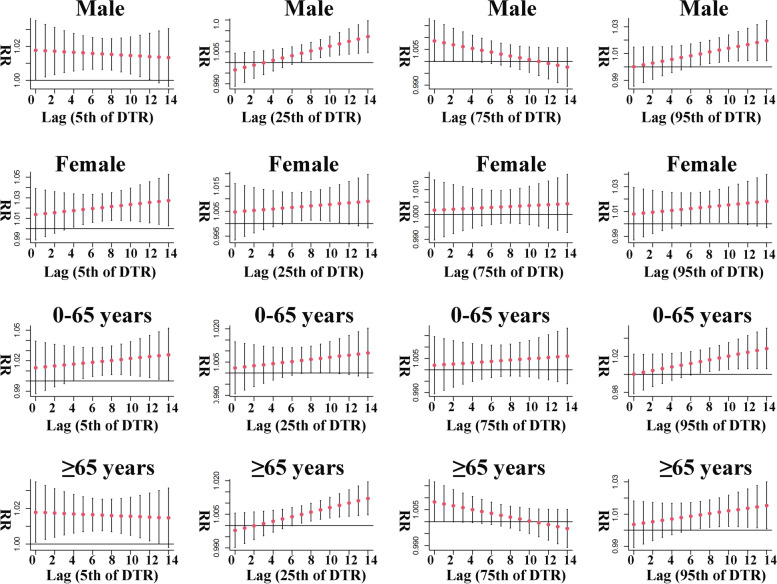
Gender and age stratified lag effect of DTR on malignant tumor mortality at various lag days

The results of the stratified risk effect of DTR and malignant tumors death are shown in Fig. 7. We found a curious phenomenon in which all four levels of DTR exposure increased the risk of malignant tumors death in male. The risk effect of low and exceedingly high levels of DTR exposure on malignant tumors mortality in male showed an increasing trend, while the risk effect of high and exceedingly low levels of DTR exposure showed a decreasing trend. According to the order of exceedingly high, high, low and exceedingly low, the maximum RR of DTR exposure for the four levels were 1.020 (95% CI: 1.005, 1.034, lag 14), 1.009 (95% CI: 1.001, 1.017, lag 0), 1.012 (95% CI: 1.005, 1.020, lag 14) and 1.018 (95% CI: 1.001, 1.035, lag 0), respectively. Only low, exceedingly high and exceedingly low DTR exposure increased the risk of death from malignant tumors in the female subgroup, which is consistent with the results of the analyses in young and elderly people. The risk of death from malignant tumors in female with DTR exposure increased, and at exceedingly high levels of exposure, the significant risk effect continued for 5 days as well as peaked at lag 11 (RR: 1.016, 95% CI: 1.001, 1.032); at low as well as exceedingly low levels of DTR exposure, the significant risk effect lasted for 6 days and 11 days, respectively, with the biggest effect at lag 11 (RR: 1.008, 95% CI: 1.001, 1.016) and lag 14 (RR: 1.027, 95% CI: 1.003, 1.053), respectively. The risk effect of DTR also tended to increase in the young people subgroup, with a maximum RR of 1.028 (95% CI: 1.006, 1.051, lag 14) for exceedingly high DTR, 1.026 (95% CI: 1.001, 1.052, lag 14) for exceedingly low DTR, and a significant effect of low DTR exposure lasting only 3 days with a maximum RR of 1.007 (95% CI: 1.001, 1.014, lag 11). The results of the elderly subgroup differed from those of the young subgroup in that the risk of malignant tumors death in the elderly showed a decreasing trend at exceedingly low DTR exposure, with the greatest RR value was 1.018, occurring on the day of exposure, and the 95% CI was 1.001 to 1.035. In addition, while both low and exceedingly high DTR exposures showed an upward trend, the risk effect appeared to be lower for low DTR with the greatest RR value was 1.007 (95% CI: 1.001, 1.020, lag 14), and exceedingly high DTR with the greatest RR value was 1.015 (95% CI: 1.001, 1.030, lag 14).

## Discussion

With the increasing attention paid to climate change and the deepening of human health research, the relationship between changes in environmental humidity as well as temperature and malignant tumors has come into notice. Although there are already studies that show the impact of climate change on chronic diseases, there are few studies on malignant tumors. In this study, we explored for the first time the short-term nonlinear correlation between meteorological factors and all malignant tumors deaths, and for the first time, AH was added for further exploration. In addition, DLNM and GAM could not consider the lag effect between variables, or the time of consideration was short, so we combined the two models and introduced the cross basis, so that the model could simultaneously describe the distribution of dependent variables in the independent variable dimension and the lag dimension, so that it could simultaneously evaluate the lag effect and nonlinear effect of exposure factors. The overall results suggest that AH, T mean, and DTR exposures all increase the all malignant tumors mortality risk. In addition, the sensitivity analysis of the model also showed that the results of the meteorological models were stable after controlling for pollutants.

The sensitivity analysis of DTR and AH showed a negative correlation (Fig. 3), which we speculate may be related to the special geographical location of Wuhu. Wuhu is located in the eastern part of inland China and has a subtropical humid monsoon climate with small diurnal temperature difference and more rainfall in summer, so DTR is negatively correlated with AH. The overall relationship between T mean, AH and DTR and the malignant tumors mortality risk is shown in Fig. 4. The results corresponding to the red line in the figure showed that exposure to these three meteorological factors increased the malignant tumors mortality risk in the population to varying degrees. Among them, both high and low DTR exposures increased the risk of malignant tumors death in the population. At present, there are few studies on meteorological factors and death from malignant tumors, and the specific influencing mechanism is still unclear. Although there are no relevant reports on the relationship between DTR and malignant tumors, a review of circadian rhythms suggested that alterations in DTR affect circadian factors in the body, allowing abnormal proliferation of human stem cells and the development of malignant tumors [19]. In addition, Sharma et al. [20] suggested that a cold environment increased the risk of death from malignant tumors because it increased the body's metabolic stress, which is consistent with our findings. In terms of humidity, our results showed that low AH increases the malignant tumors mortality risk. Disappointingly, there are also no relevant reports on the relationship between humidity and malignant tumors. However, in a study of the relevance of geography to breast cancer in Iran, we found that high regional humidity increased the short-wave reflection of sunlight, thereby reducing people's exposure to the electromagnetic spectrum, while low humidity exposure significantly increased the incidence of malignant tumors [8]. Solar radiation has been shown to increase malignant tumors death risk [21], so we speculated that increased solar radiation in low-humidity environments may be one of the reasons for increased malignant tumors mortality.

The relationship between specific AH and death from malignant tumors is shown in Table 2. The results showed that short-term exposure to low and exceedingly low AH increased the malignant tumors mortality risk. In addition to the above mentioned humidity affecting outdoor radiation content, we also found a study on humidity and skin, which showed that the increase in humidity would increase the water content of human skin cuticle, thus reducing the amount of radiation exposure to skin and dry skin symptoms [22]. It is well known that many radiations can cause malignant tumors, and solar radiation is classified as one of the occupational carcinogens. Many studies have been reported that radiation from sunlight can cause many types of malignant tumors, such as skin cancers [23], thyroid cancers [24], lung cancers [25], lip cancers [26]. Both low and exceedingly low levels of AH exposure increased the malignant tumors mortality risk in this study, and the risk effect value of exceedingly low AH was higher than that of low AH. Therefore, we believed that low level of AH exposure increased the amount of solar radiation exposure in the population, thus increasing the malignant tumors mortality risk. The stratified analysis results of AH are shown in Fig. 5. Results showed an increased malignant tumors mortality risk in male, female, and young adults, but not in the elderly. The malignant tumors mortality risk in male was increased only at exceedingly low AH exposure and lasted for 7 days from lag 4. However, female seemed to be more sensitive to the effects of AH, and not only was the risk of death from malignant tumors increased at low and exceedingly low AH exposures, but this risk was actually reduced at high AH exposures. From this, it can be further inferred that solar radiation may have a greater effect on female than male. Interestingly enough, we observed that AH exposure affects young people more than female: at low and exceedingly low AH exposure, the maximum RR values for malignant tumors death in young people were 1.022 and 1.035 (1.016 and 1.018 for female), respectively, and the duration was 13 and 13 days (8 and 5 days for female), respectively. In addition, we also observed that there was no significant effect of all levels of AH exposure on the risk of death in the elderly. We believed that these phenomena were not difficult to explain, because compared to young people, the elderly spend less time outdoors, so that are exposed to less solar radiation, and the majority of female population with malignant tumors deaths are elderly [27], which explained why AH exposure did not have a significant effect on the elderly, as well as the female was less affected than the young.

Table 3 demonstrates the relationship between specific T mean and malignant tumors mortality. Similar to the results for AH, the malignant tumors mortality risk in the population was significantly increased with low and exceedingly low T mean exposure. Many studies have shown that low temperature had a significant impact on lung cancer [28], breast cancer [8], leukemia [29] and other malignant tumors. A study of environmental risk of death in Hong Kong [30] showed that exposure to cold environments increased the risk of death from malignant tumors in the population, which is consistent with our findings. We speculate that this phenomenon may be related to cold stress in the human body in the cold state. Shreetama et al. [29] suggested that cold exposure affected the expression of certain genes in the body, such as heat shock proteins and uncoupling proteins were elevated, and in biochemicals, cholesterol, norepinephrine and thyroxine were also elevated. Abe et al. [31] suggested that cold increases the activity of brown adipose tissue in humans. In addition, other reports have demonstrated that cold causes mutations in tumor suppressor genes such as p53 and BRCA1 [32, 33]. All the above stressful phenomena may lead to the aggravation of malignant tumors or even death. In the stratified analysis, all strata had an increased risk of death from malignant tumors at low and exceedingly low T mean exposures (Fig. 6). Female risk of death was higher than male at both levels of T mean exposure, suggesting a stronger cold stress response in female than male. However, young people appear to be more sensitive to cold exposure, as only young people show an upward trend in the risk of malignant tumors death at low and exceedingly low T mean exposures, suggesting that the deleterious effects of cold on young people increase progressively over time. It has been reported that heat sensitivity in humans decreases with age [34], moreover, young people had a stronger immune system and body regulation than older people, which also explains the reduced risk of malignant tumors in young people with high T mean exposure. According to one study [35], cold stress by temperature changes could precipitate sudden health changes in sick cancer patients, affect cardiorespiratory system and could also affect malignant tumor patients on chemotherapy treatment. This could also increase the risk of death from malignant tumors in the population. Additionally, snowfall accompanied by cold weather could also have a certain impact on traffic under low temperature, which made people unable to seek medical treatment in time when they were sick, thus leading to the highest risk of death when cold weather comes. Because there is less rainfall in winter, the water vapor content in the air is lower. According to the above analysis results, low T mean and AH will increase the risk of death from malignant tumors. Therefore, we also guessed that the risk of death from malignant tumors may also be related to the season. One study shows that the prognosis of malignant tumors deteriorates in winter, while the prognosis is better in summer [36]. Another study found that malignant tumors had the highest mortality rate in the winter [37]. These results are consistent with our guess.

An interesting finding in our results for specific DTR is that all four grades of DTR exposure increase the risk of malignant tumors death in the total population (Table 4). This indicates that DTR exposure at the 50th percentile level (9 ℃) has the lowest risk of death from malignant tumors in the population compared to the four levels in the table. We believed that low DTR exposure and high DTR exposure are two opposite exposure states, so they do not have the same mechanism of effect in increasing malignant tumors, therefore, we divided the following explanation into two points to elaborate. First, in humid subtropical monsoon climates, the season in which low DTR occurs tends to be in winter [38], suggesting that the mechanism by which low DTR exposure increases the malignant tumors mortality risk may be the same as that of cold exposure above. Second, since there are no mechanistic studies on DTR and malignant tumors, we reviewed other literature and speculated that high DTR can increase malignant tumors mortality risk by affecting human stem cells functions; the higher the DTR, the more likely it is to affect one's circadian rhythm, and one study suggested that changes in circadian rhythm may increase malignant tumor death risk by affecting the functions of the human clock genes CLOCK and BMAL1 [19]. Yu et al. [39] suggested that damage to the clock gene could accelerate the proliferation of HCT116 cells in colorectal malignant tumor cells, thereby increasing the death risk of disease. Dierickx et al. [40] suggested that alterations in clock genes CLOCK and BMAL1 affect the circadian network of cardiomyocytes, and that altered rhythmicity of cardiomyocytes exacerbates the toxic side effects of doxorubicin, an anticancer drug that is widely used, on the heart [41]. All these results confirm our suspicions. Figure 7 shows the results for gender and age stratification. Results showed an increased malignant tumors mortality risk in female, young and elderly with low, exceedingly low and exceedingly high DTR exposure. However, the malignant tumors mortality risk in male increased at all four levels (low, exceedingly low, high, exceedingly high) of DTR exposure. This suggested that male appear to be more sensitive to high DTR exposure.

In this study, the effects of air pollutants were controlled by DLNM and GAM, and the relationship between AH, T mean, DTR and death from malignant tumor was evaluated by stratification of gender and age. However, there are some limitations of our study. Firstly, in this study we used data from monitoring sites rather than the true exposure levels of individuals, which, similar to other ecological studies, may misclassify exposure levels. Secondly, this study only included air pollutants as confounding factors without controlling for other potential factors, such as economic status, dietary habits and lifestyle of patients with malignant tumors, making some risk effects less pronounced and possibly leading to limited biological significance. Thirdly, the present study was designed to examine the short-term effects of meteorological factors on the risk of death from malignant tumors, which may have led us to overlook the long-term effects between the two. Therefore, the long-term exposure to meteorological factors needed to be evaluated in the follow-up studies. Fourthly, the area of this study was limited to Wuhu City, which meant that more areas were needed for the extension study. Fifthly, since this study is a study on the risk of death of all malignant tumors, and the proportion of mortality of each tumor type cannot be guaranteed to be completely consistent, resulting in relative risk may not be a clinically significant change, though is statistically significant. For example, changes in temperature and humidity may largely affect the respiratory system, leading to lung cancer-related deaths. However, few studies have been reported on the topic and hence unable to ascertain that if there is a true effect of meteorological factors on other malignant tumors (other than lung cancer). Finally, more mechanistic studies are needed to clarify the relationship between meteorological factors and the risk of death from malignant tumors.

In conclusion, the global climate is changing as society advances and develops, and the resulting disease burden is increasing. Malignant tumors, as the number one deadly disease among Chinese urban residents, have received strong attention. As the first study to explore the relationship between meteorological factors and all-malignant tumors death, the results of this study are intended to provide suggestions for future research in this field, and also provide certain reference value for local governments to make policies. Therefore, more exploratory and mechanistic researches are needed to address the impacts of climate change on human health. At the same time, local governments also need to adjust population health policies according to the season and other factors to reduce the risk of death from malignant tumors.

## Conclusions

The results of this study showed that AH, T mean and DTR all increased the risk of malignant tumors death in the Wuhu population. Female appear to be more sensitive to humidity, while male require additional attention to reduce exposure to high level of DTR.

## Data Availability

The datasets analysed during the current study is available from the corresponding author on reasonable request.
